# Genome-Wide Identification and Co-Expression Analysis of *ARF* and *IAA* Family Genes in *Euscaphis konishii*: Potential Regulators of Triterpenoids and Anthocyanin Biosynthesis

**DOI:** 10.3389/fgene.2021.737293

**Published:** 2022-01-05

**Authors:** Bobin Liu, Juanli Zhu, Lina Lin, Qixin Yang, Bangping Hu, Qingying Wang, Xiao-Xing Zou, Shuang-Quan Zou

**Affiliations:** ^1^ Jiangsu Key Laboratory for Bioresources of Saline Soils, Jiangsu Synthetic Innovation Center for Coastal Bio-agriculture, School of Wetlands, Yancheng Teachers University, Yancheng, China; ^2^ College of Forestry, Fujian Agriculture and Forestry University, Fuzhou, China; ^3^ Fujian Colleges and Universities Engineering Research Institute for Conservation and Utilization of Natural Bioresources, Fuzhou, China

**Keywords:** *Euscaphis konishii*, Aux/IAA, ARF, triterpenoids, anthocyanin, fruit development

## Abstract

*Euscaphis konishii* is an evergreen plant that is widely planted as an industrial crop in Southern China. It produces red fruits with abundant secondary metabolites, giving *E*. *konishii* high medicinal and ornamental value. Auxin signaling mediated by members of the AUXIN RESPONSE FACTOR (ARF) and auxin/indole-3-acetic acid (Aux/IAA) protein families plays important roles during plant growth and development. *Aux/IAA* and *ARF* genes have been described in many plants but have not yet been described in *E. konishii*. In this study, we identified 34 EkIAA and 29 EkARF proteins encoded by the *E. konishii* genome through database searching using HMMER. We also performed a bioinformatic characterization of *EkIAA* and *EkARF* genes, including their phylogenetic relationships, gene structures, chromosomal distribution, and *cis*-element analysis, as well as conserved motifs in the proteins. Our results suggest that *EkIAA* and *EkARF* genes have been relatively conserved over evolutionary history. Furthermore, we conducted expression and co-expression analyses of *EkIAA* and *EkARF* genes in leaves, branches, and fruits, which identified a subset of seven *EkARF* genes as potential regulators of triterpenoids and anthocyanin biosynthesis. RT-qPCR, yeast one-hybrid, and transient expression analyses showed that EkARF5.1 can directly interact with auxin response elements and regulate downstream gene expression. Our results may pave the way to elucidating the function of *EkIAA* and *EkARF* gene families in *E. konishii*, laying a foundation for further research on high-yielding industrial products and *E. konishii* breeding.

## Introduction

Plant secondary metabolites are not only important to plant development, but are also significant as nutritional resources for humans, as sources for color and odorants, and as potential materials for drug discovery (Huang et al., 2020a; Bing et al., 2021). Secondary metabolites include terpenes, phenolic compounds, and alkaloids, which play essential roles as food additives and in medicine, cosmetics, skincare, and industrial chemicals. The biosynthesis of plant secondary metabolites is very complex and is regulated by various environmental signals and development cues. Because of their critical roles, much attention is being paid to the biosynthesis and regulatory mechanism of secondary metabolites.

The phytohormone auxin regulates a wide range of processes in plant growth and development, including vascular differentiation, lateral root formation, apical dominance, and fruit development (Liscum and Reed, 2002; Aloni et al., 2006). The auxin/indole-3-acetic acid (Aux/IAA) and AUXIN RESPONSE FACTOR (ARF) proteins are critical players of auxin signal transduction (Hagen, 2015). ARFs are transcription factors consisting of a DNA-binding domain (DBD), a middle transcriptional regulatory region (MR), and a dimerization domain at their C termini (CTD), with the MR serving as an activation or repressor domain (Tiwari et al., 2003; Guilfoyle and Hagen, 2007). ARF DBDs bind to auxin response elements (AuxREs) located in the promoter region of auxin-responsive genes to activate or repress their transcription, depending on the type of MR (Tiwari et al., 2003; Guilfoyle and Hagen, 2007). Aux/IAA proteins comprise four domains, denoted domains I, II, III, and IV (Tiwari et al., 2001). Located at the N terminus, domain I is characterized by the LxLxLx motif and is mainly responsible for repression of gene expression. Domain II mediates the degradation of the protein via the degron sequence (GWPPV) by the 26S proteasome (Hagen and Guilfoyle, 2002; Tiwari et al., 2004). Domains III and IV at the C terminus interact with the CTD of ARFs to form homo- and heterodimers, resulting in the transcriptional induction or repression of downstream auxin-responsive genes (Ulmasov et al., 1999; Guilfoyle, 2015). The interaction between Aux/IAA and ARF proteins also depends on auxin concentration. When auxin levels are low, ARFs bind to AuxREs, but transcription is repressed through their interaction with Aux/IAAs. With increasing auxin levels, Aux/IAAs are degraded via the 26S proteasome, thus alleviating the repressive effect of Aux/IAAs on ARFs (Gray et al., 2001; Winkler et al., 2017; Roosjen et al., 2018). Both Aux/IAA and ARF proteins are encoded by large gene families with distinct expression patterns and regulatory mechanisms, contributing to the complexity of auxin signaling (Luo et al., 2018).

Aux/IAA and ARF family members play critical and extensive roles during the entire plant life cycle. During embryogenesis, ARF5 is involved in organ formation in Arabidopsis (*Arabidopsis thaliana*) (Hardtke and Berleth, 1998). ARF19 and ARF7 redundantly regulate lateral root initiation (Wilmoth et al., 2005), while ARF1 and ARF2 control floral organ senescence and abscission (Ellis et al., 2005). Most of the Aux/IAA family members described in Arabidopsis, such as IAA3, IAA14, IAA28, and IAA19, affect the growth and development of lateral roots (Goh et al., 2012; Luo et al., 2018). Aux/IAAs and ARFs also play important roles in regulating fruit development. ARF8 was reported to play a negative role during fertilization and fruit initiation in Arabidopsis (Goetz et al., 2006). SlARF10 and SIARF6A are involved in chlorophyll and sugar accumulation during tomato (*Solanum lycopersicum*) fruit development (Yuan et al., 2018a; Yuan et al., 2019). Auxin signaling also interacts with other phytohormone signaling pathways to control fruit development. For example, the tomato gibberellic acid (GA) repressor SlDELLA interacts with SlARF7/SlIAA9 to regulate fruit initiation (Hu et al., 2018). Likewise, SlIAA3 contributes to differential growth by integrating auxin and ethylene signaling (Chaabouni et al., 2009). The accumulation of secondary metabolites is an important biological process during fruit ripening, which includes fruit color formation and biosynthesis of volatile odorants. In apple (*Malus domestica*), MdARF13 interacts with MdIAA121 to regulate anthocyanin biosynthesis (Wang et al., 2018). Overexpression of *MdIAA26* boosts anthocyanin biosynthesis in apple and Arabidopsis seedlings (Wang et al., 2020). In addition to anthocyanins, auxin induces the accumulation of flavonols by promoting the expression of the gene encoding a key biosynthetic enzyme (Lewis et al., 2011). Auxin signaling also participates in the accumulation of glucosinolates, a class of important plant defense metabolites (Mitreiter and Gigolashvili, 2021). Although auxin plays critical roles in secondary metabolite biosynthesis, how auxin signaling synergistically regulates the contents of multiple secondary metabolites is unclear.

*Euscaphis* plants belong to the Staphyleaceae family and are deciduous shrubs or small trees widely distributed in East Asia, from Japan to Southern China (Zhang et al., 2012). These plants constitute an important industrial crop due to the medicinal compounds and other industrially desirable products extracted from their fruits, leaves, and roots, such as triterpenes, phenolic acid, and flavonoids (Liang et al., 2018). In China, *E. konishii* is cultivated as an ornamental plant due to its beautiful red-winged pericarp and also has a long history of use as a medicinal plant to cure colds and allergies (Yuan et al., 2018b). The potential medical applications of *Euscaphis* were recently supported by data indicating that total phenolic and methanolic extracts of *Euscaphis* plants can mitigate liver fibrosis in mice and inhibit hepatic stem cell proliferation (Lee et al., 2009; Huang et al., 2020b), prompting the expansion of *E. konishii* cultivation (Sun et al., 2019). Therefore, *E. konishii* is an economically useful crop for the production of medicinal compounds. However, the molecular mechanisms governing the biosynthesis of these compounds in this species are poorly understood, which limits the breeding of improved *E. konishii* varieties. Although the Aux/IAA and ARF families have been systematically identified and characterized in many plants, including Arabidopsis (Remington et al., 2004; Okushima et al., 2005), rice (*Oryza sativa*) (Jain et al., 2006; Wang et al., 2007), poplar (*Populus trichocarpa*) (Kalluri et al., 2007), maize (*Zea mays*) (Wang et al., 2010; Xing et al., 2011), soybean (*Glycine max*) (Van Ha et al., 2013; Singh and Jain, 2015), and pepper (*Capsicum annuum*) (Yu et al., 2017; Waseem et al., 2018), their functions in *E. konishii* are not clear. Given the importance of Aux/IAA and ARF proteins in plant development, we undertook a comprehensive survey of the *Aux/IAA* and *ARF* gene families in *E. konishii* to better understand the biosynthetic pathways of medicinal secondary metabolites. In this study, we identified 34 *Aux/IAA* and 29 *ARF* genes in *E. konishii* and analyzed their sequence features, phylogenetic relationships, *cis*-elements, and co-expression profiles. We also explored the function of EkARFs in the context of phenolic and anthocyanin biosynthesis. Our results may have uncovered a potential role for auxin in the biosynthesis of secondary products, which may provide useful information for breeding *E. konishii* with a high content of medicine compounds.

## Materials and Methods

### Identification of *IAA* and *ARF* Genes

Hidden Markov model (HMM) logos of Aux/IAA (PF02309) and ARF (PF06507) proteins were downloaded from the Pfam database (Finn et al., 2014) and used to scan the *E. konishii* predicted proteome (Sun et al., 2021) with the HMMER software package (Finn et al., 2011). The resulting Aux/IAA and ARF candidates were further used to generate HMM logos for EkIAAs and EkARFs using hmm-build from the HMMER suite (Finn et al., 2011), before scanning the *E. konishii* proteome again. Proteins with an *E*-value lower than 0.01 were retained, and the presence of conserved ARF or IAA domains was confirmed using the Conserved Domains Database (Marchler-Bauer et al., 2011), Pfam (Finn et al., 2014), and the Simple Modular Architecture Research Tool (Letunic and Bork, 2018). The proteins meeting all of the above criteria were used for further study. The number of amino acids, the predicted molecular weight, and the theoretical isoelectric point (pI) were determined using the ExPASy server (http://web.expasy.org/protparam/) (Gasteiger et al., 2003).

### Gene Structure and Motif Analysis

TBtools (Chen et al., 2020) was employed to illustrate the exon/intron structures of all *EkIAA* and *EkARF* genes. Conserved protein motifs in their encoded proteins were predicted by the MEME program (parameters: number of maximum patterns, 10; maximum width, 50) (http://memesuite.org/tools/meme) (Bailey et al., 2006).

### Multiple Sequence Alignment and Phylogenetic Analysis

The protein sequences for the 29 ARFs and 34 IAAs from Arabidopsis were obtained from published references (https://www.arabidopsis.org), and the protein sequences of 14 ARFs and 11 IAAs from *Amborella trichopoda* were identified following the same method described for *E. konishii*. Full-length protein sequences for all IAAs and ARFs identified in *E. konishii*, Arabidopsis, and *A. trichopoda* were used for phylogenetic analysis. The phylogenetic tree was built with the maximum likelihood method on the IQ-TREE web server (http://iqtree.cibiv.univie.ac.at/) (Nguyen et al., 2015).

### Analysis of *Cis*-acting Elements in the *EkIAA* and *EkARF* Promoters

The upstream sequences (2 kb) of *EkIAA* and *EkARF* genes were extracted via TBtools (Chen et al., 2020) and then submitted to the PlantCARE database (Lescot et al., 2002) (http://bioinformatics.psb.ugent.be/webtools/plantcare/html/) to identify *cis*-elements.

### *EkIAA* and *EkARF* Chromosomal Location and Duplication Event Analysis

The chromosomal distribution and location of all *EkIAA* and *EkARF* genes were acquired from the *E. konishii* genome annotation file. Colinear circles for *EkIAA* and *EkARF* genes were drawn with TBtools (Chen et al., 2020). Duplication events were confirmed on the basis of coverage (>70% of the entire gene body) and similarity (70%) of the two aligned sequences (Gu et al., 2002) and were considered tandem duplication pairs if they were located within 100 kb (Mehan et al., 2004). Genes located in duplicated regions with 70% similarity were identified as segmental duplications (Mehan et al., 2004). *K*_a_/*K*_s_ values were calculated with TBtools (Chen et al., 2020).

### Expression Analysis of *IAA* and *ARF* Genes by Transcriptome Deep Sequencing (RNA-Seq)

The RNA-seq data for three development stages (green, turning, and red fruit) and three tissues (red-winged pericarp, branch, and leaf) were downloaded from the National Center for Biotechnological Information (NCBI) Sequence Read Archive (SRA) under the accession numbers PRJNA548305 and PRJNA548305, respectively. The RNA-seq reads were mapped to the *E. konishii* reference genome via Salmon algorithm, and the transcripts per million reads (TPM) for *AUX/IAA* and *ARF* genes were extracted for further analysis. The heatmaps were drawn using TBtools (Chen et al., 2020).

### Correlation Analysis

To study the effect of auxin on the regulation of anthocyanin and terpenoid biosynthesis in *E. konishii*, a comprehensive correlation analysis was first performed using the correlation test in R between anthocyanin contents and the expression levels of anthocyanin biosynthetic genes. Biosynthetic genes whose expression was positively correlated with anthocyanin contents were selected for further correlation analysis between their expression levels and those of *EkARF* genes. Pearson’s correlation coefficients (*r*, *p*-value < 0.05) were used to define five correlation levels: no correlation (|*r*| ≤ 0.2), weak correlation (0.21 ≤ |*r*| ≤ 0.35), moderate correlation (0.36 ≤ |*r*| ≤ 0.67), strong correlation (0.68 ≤ |*r*| ≤ 0.90), and very strong correlation (0.91 ≤ |*r*| ≤ 1), with *r* > 0 indicating positive correlations and *r* < 0 negative correlations (Prion and Haerling, 2014).

### RT-qPCR Verification of *EkARF5.1* Gene Expression

Fruits at the green stage, turning stage, and red stage from *E. konishii* were harvested as materials for RT-qPCR. Total RNA was extracted using the RNAprep Pure kit (Tiangen, China), and then 1 µg of total RNA per sample was subjected to reverse transcription using the PrimeScript RT Reagent Kit (Takara, China) with gDNA Eraser (Takara, China). The specific primers for *EkARF5.1* (F: 5′-GCA​ACC​TCC​AAC​TCA​AGA​GC-3′, R: 5′-GAC​GCC​TCA​CAC​CCA​CTA​AT-3′) were designed by Primer 5 software and synthetized by Sangon Biotech (Shanghai, China). *UBC23* (F: 5′-AGC​CAC​ATA​ATC​TCC​GTG​TAA​G-3′, R: 5′-GCT​GAC​CAT​GTT​CGA​GTA​GTT-3′) was used as an internal reference (Yuan et al., 2018b). The reaction mixture consisted of 10 µl of 2× GoTaq qPCR Master Mix (Promega, United States), 0.4 µl of each gene-specific primer, 1 µl of cDNA (10× dilution), 0.4 µl of dye, and 7.8 µl of nuclease-free water. The reaction conditions were as follows: 95°C for 2 min followed by 40 cycles of 95°C for 15 s and 60°C for 1 min. Relative gene expression levels were calculated by the comparative ΔCt method. Three biological replications were assessed per sample.

### DNA Binding and Transactivation Assay

The full-length *EkARF5.1* coding sequence was cloned into the pGBKT7 vector to generate BD-EkARF5.1, which was then introduced into yeast strain Y2HGold. The resulting colonies were grown on synthetic defined (SD) medium lacking Trp and His for 2 days to observe the transcriptional activation activity of EkARF5.1, using empty pGBKT7 as a negative control.

To test the binding of EkARF5.1 to AuxREs, seven repeats of the AuxRE element (TGTCTC) were inserted into the multiple cloning site of the pAbAi vector to generate the 7×TGTCTC-pAbAi vector, which was integrated into the Y1HGold genome to construct the bait reporter strain. The AD-EkARF5.1 clone was generated by subcloning the full-length *EkARF5.1* coding sequence into the pGADT7 vector. AD-EkARF5.1 was then transformed into the bait reporter strain. The transformants were spotted onto SD medium lacking Leu or the same medium containing 100 ng/ml of the antibiotic aureobasidin A (AbA) and allowed to grow for 48 h to analyze binding activity.

Constructs consisting of the *β-GLUCURONIDASE* (*GUS*) reporter *7*×*TGTCTC*:*GUS* (cloned in pMDC164) and *35S*:*EkARF5.1* (cloned in pDMC32) were introduced into Agrobacterium (*Agrobacterium tumefaciens*) strain GV3101 for infiltration in *Nicotiana benthamiana* leaf epidermal cells. Agrobacterium harboring *7*×*TGTCTC*:*GUS* and *35S*:*EkARF5.1* were infiltrated into the abaxial side of *N. benthamiana* leaves with a syringe as described previously (Liu et al., 2014). *N. benthamiana* leaves were stained for GUS activity 3 days after infiltration.

### Data Availability Statement

Publicly available datasets were analyzed in this study. This data can be found here: The E. konishii chromosome-level genome assembly and annotation data (Accession No. PRJCA005268/GWHBCHS00000000) were available from National Genomics Data Center at BioProject/GWH (https://bigd.big.ac.cn/gwh c).

## Results

### Genome-wide Identification of *EkIAA* and *EkARF* Genes in the *E. konishii* Genome

**FIGURE 1:**
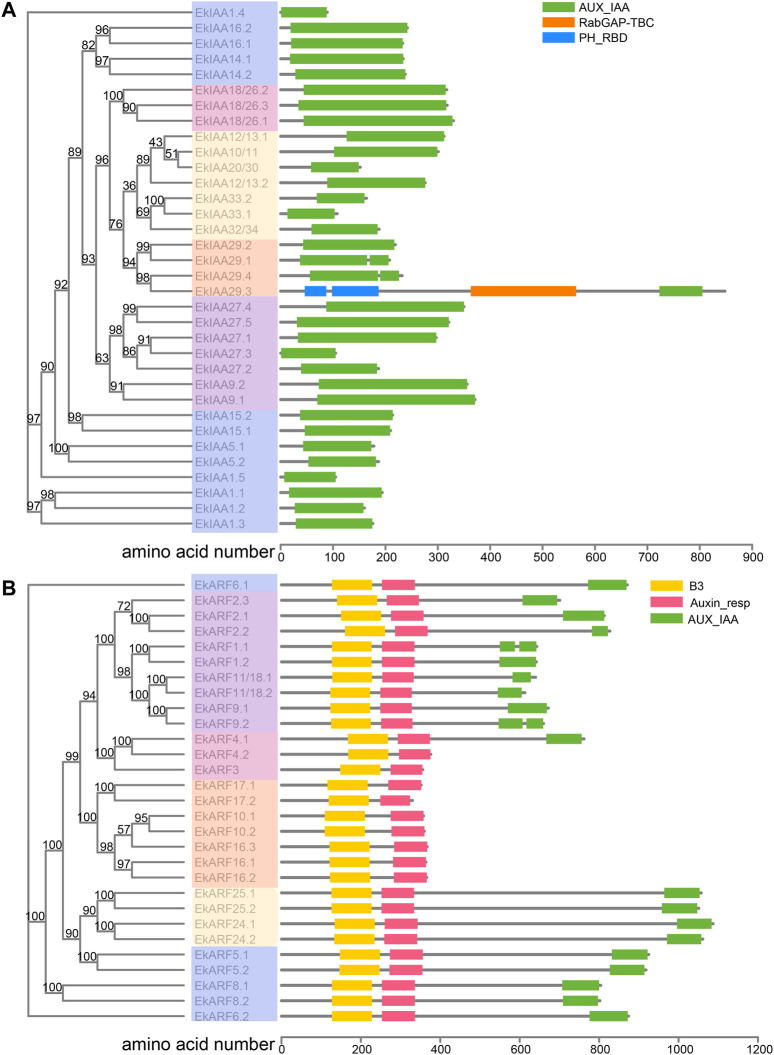
Phylogenetic relationships and protein domain maps of *E. konishii* IAA **(A)** and ARF **(B)** proteins. Left, phylogenetic relationship of 34 *IAAs* and 29 *ARFs* from *E. konishii*. Different colors represent different groups. Right, IAA and ARF protein domain composition. Bootstrap values are shown close to branch nodes.

To identify *EkIAA* and *EkARF* genes, we searched the *E. konishii* genome using HMMER v3 in two rounds (see Materials and Methods for details). We analyzed the resulting protein sequences using the Conserved Domains Database, Simple Modular Architecture Research Tool, and Pfam database, which resulted in 34 Aux/IAA and 29 ARF candidate proteins (Figure 1; Supplementary Table S1). We numbered the *E. konishii IAA* and *ARF* genes based on their homologs in Arabidopsis (Remington et al., 2004; Okushima et al., 2005); the full list is provided in Supplementary Table S1, along with their gene IDs, their coding sequences, genomic DNA and protein sequences, the lengths of the coding and protein sequences, and the predicted molecular weights and isoelectric points (pI) of the proteins.

We observed a broad variation in the lengths and biochemical properties of EkIAA and EkARF proteins. EkIAA proteins ranged from 91 (EkIAA1.4) to 849 (EkIAA29.3) amino acids, with predicted molecular weights from 10.5 to 97.2 kDa (Supplementary Table S1). The predicted pI values of EkIAA proteins varied from 4.7 (EkIAA32/34) to 9.3 (EkIAA33.2). Similarly, EkARF proteins ranged in length from 432 (EkARF17.2) to 1,110 (EkARF24.1) amino acids with predicted molecular weights from 48.2 to 122.7 kDa (Supplementary Table S1). The predicted pI values of EkARF proteins varied from 5.41 (EkARF5.1) to 8.42 (EkARF10.2) (Supplementary Table S1).

### Phylogenetic Analysis of EkIAAs and EkARFs

**FIGURE 2:**
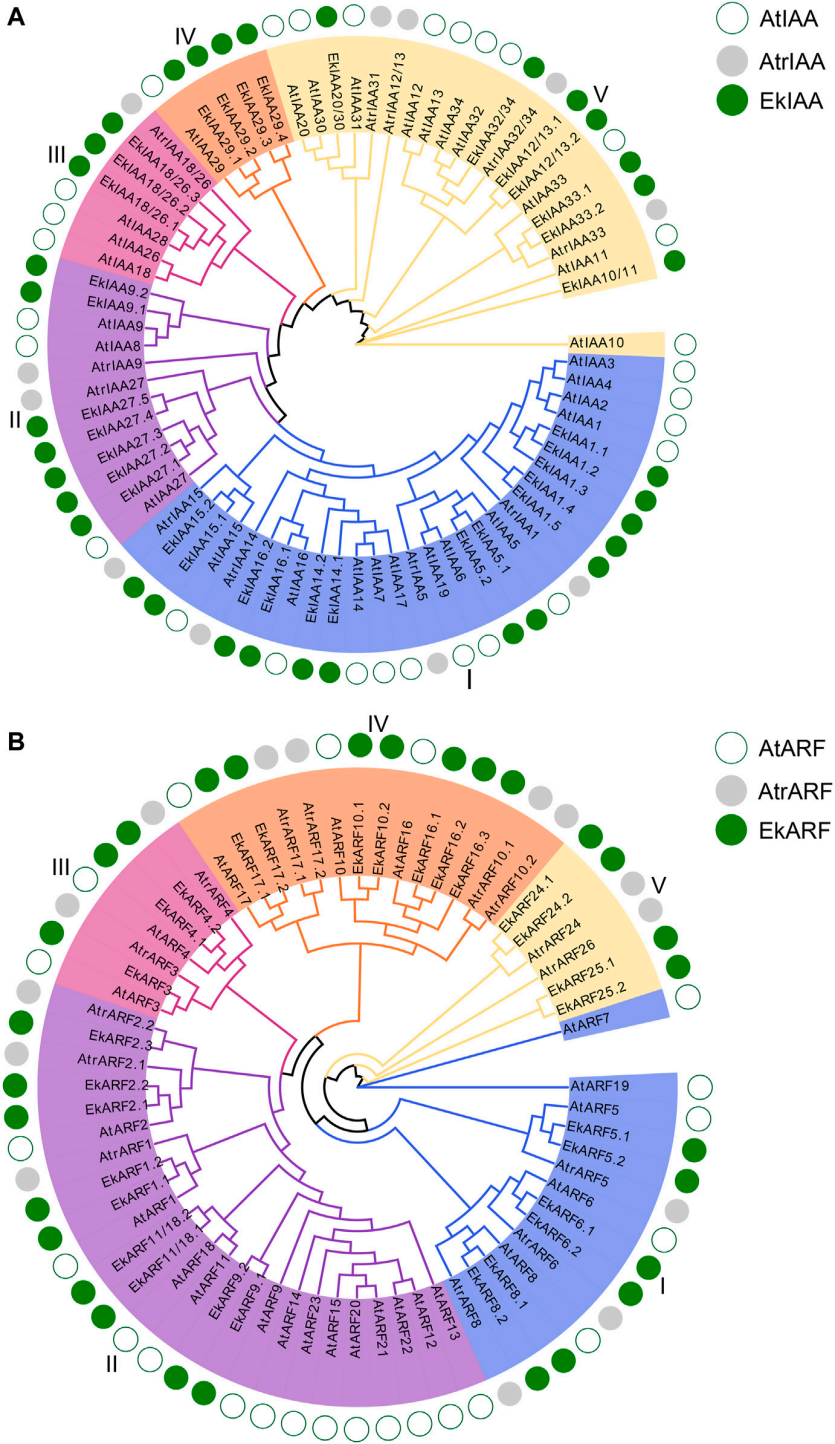
Phylogenetic trees of EkIAAs **(A)** and EkARFs **(B)** from Arabidopsis, *Amborella trichopoda*, and *E. konishii*. Different groups have different colors. The green open circles, gray circles, and green solid circles represent Arabidopsis, *A. trichopoda*, and *E. konishii*, respectively.

To better understand their evolutionary history, we subjected all IAAs and ARFs identified in the model plant Arabidopsis, the early angiosperm *A. trichopoda*, and *E. konishii* to phylogenetic analysis with the MEGA-X software package (Kumar et al., 2018) and the IQ-TREE web server (Nguyen et al., 2015). In both protein families, individual members clustered into five branches, indicating that IAAs and ARFs are highly differentiated (Figure 2A), as previously reported in Arabidopsis (Remington et al., 2004) and poplar (Kalluri et al., 2007). EkIAA proteins were equally divided among groups Ⅰ, Ⅲ, and Ⅴ (Figure 2A). *EkIAA5* and *EkIAA15* from group I appeared to have undergone gene duplication, while group I had no clear *E. konishii* orthologs for Arabidopsis *IAA6* or *IAA19* (Figure 2A). As illustrated by the size of groups Ⅱ and Ⅳ, *EkIAA* genes have undergone gene duplication, especially *IAA27* and *IAA29* (Figure 2A). As with IAAs, the phylogenetic analysis of ARFs also divided the proteins into five groups (Figure 2B), as previously reported in Arabidopsis (Okushima et al., 2005). Group I consisted of six *EkARF* and five Arabidopsis *ARF* members, the latter having been reported to exhibit transcriptional activation activity (Guilfoyle and Hagen, 2007). Of note, *ARF5*, *ARF6*, and *ARF8* all showed gene duplication in the *E. konishii* genome, while orthologs for *ARF7* and *ARF19* appeared to be lacking. Some, but not all, group II members showed signs of gene duplication (Figure 2B), for example, *ARF1* and *ARF9*. Notably, IAAs and ARFs displayed the same distribution across groups in Arabidopsis and *E. konishii*, with the exception of group Ⅴ ARFs, which indicates that the *IAA* and *ARF* gene families in *E. konishii* are likely conserved. Within each group, several *EkIAA* and *EkARF* members had experienced duplication, with the exception of group Ⅴ IAA members (Figure 2A). In addition, we noted the absence of clear orthologs for several IAAs and ARFs in several groups (Figure 2), indicative of their independent evolution in *E. konishii* since the divergence from Arabidopsis.

### Chromosomal Location and Gene Duplication Events of *EkIAA* and *EkARF* Genes

**FIGURE 3:**
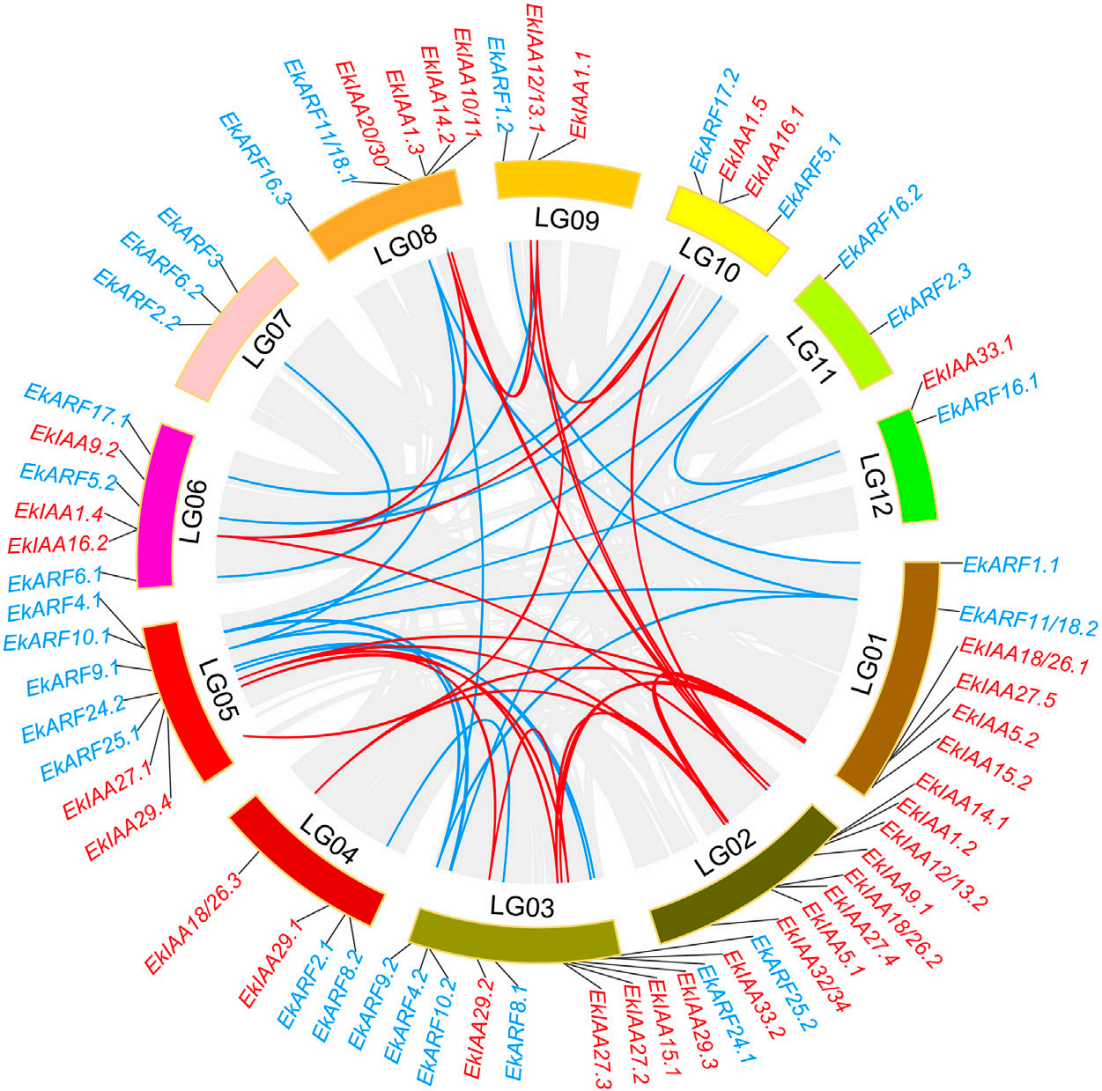
Chromosomal locations and segmental duplicated genes for 34 *EkIAA* and 29 *EkARF* genes. Linkage group numbers (LG01–LG12) are shown at the bottom. Gray lines show all synteny blocks in the *E. konishii* genome, red lines show segmental duplication of *IAA* genes, and blue lines show segmental duplication of *ARF* genes.

We determined the genomic positions of all *EkIAA* and *EkARF* genes along the linkage groups (LGs) of the *E. konishii* genome. Both groups of genes were unevenly distributed in the *E. konishii* LGs (Figure 3). For example, LG02 alone harbored eight *EkIAA* genes, whereas no *EkIAA* gene mapped to LG07 or LG11 (Figure 3). Several *EkIAA* genes clustered in close proximity on LG01, LG2, LG03, and LG08 (Figure 3). The pattern for the *EkARF* genes was similar: six *EkARF* genes mapped to LG03, five to LG05, three each to LG06 and LG07, one each to LG09 and LG12, and none to LG02 (Figure 3).

During genome evolution, gene duplication and neofunctionalization are driven by tandem and segmental duplication (Cannon et al., 2004; Freeling, 2009). To elucidate the expansion of the *EkIAA* and *EkARF* gene families in *E. konishii*, we studied their segmental and tandem duplications*.* We identified 20 instances of segmental duplication events, involving 11 *EkIAA* and 20 *EkARF* genes, but no tandem duplication events in either gene family (Figure 3). For the 10 pairs of segmental duplicated *EkIAA* genes and the 10 pairs of segmental duplicated *EkARF* genes identified above, we calculated the ratios of nonsynonymous to synonymous substitutions (*K*_a_/*K*_s_) to evaluate their molecular evolutionary rates (Supplementary Table S2). The ratios for all duplicated pairs were less than 1 (Supplementary Table S2), suggesting that duplicated pairs of genes underwent purifying selection during evolution, thus raising the possibility that the biochemical characteristics of these EkIAAs and EkIAAs may not have changed very much since the initial duplication event.

### Analysis of Conserved Motifs and Gene Structure

**FIGURE 4:**
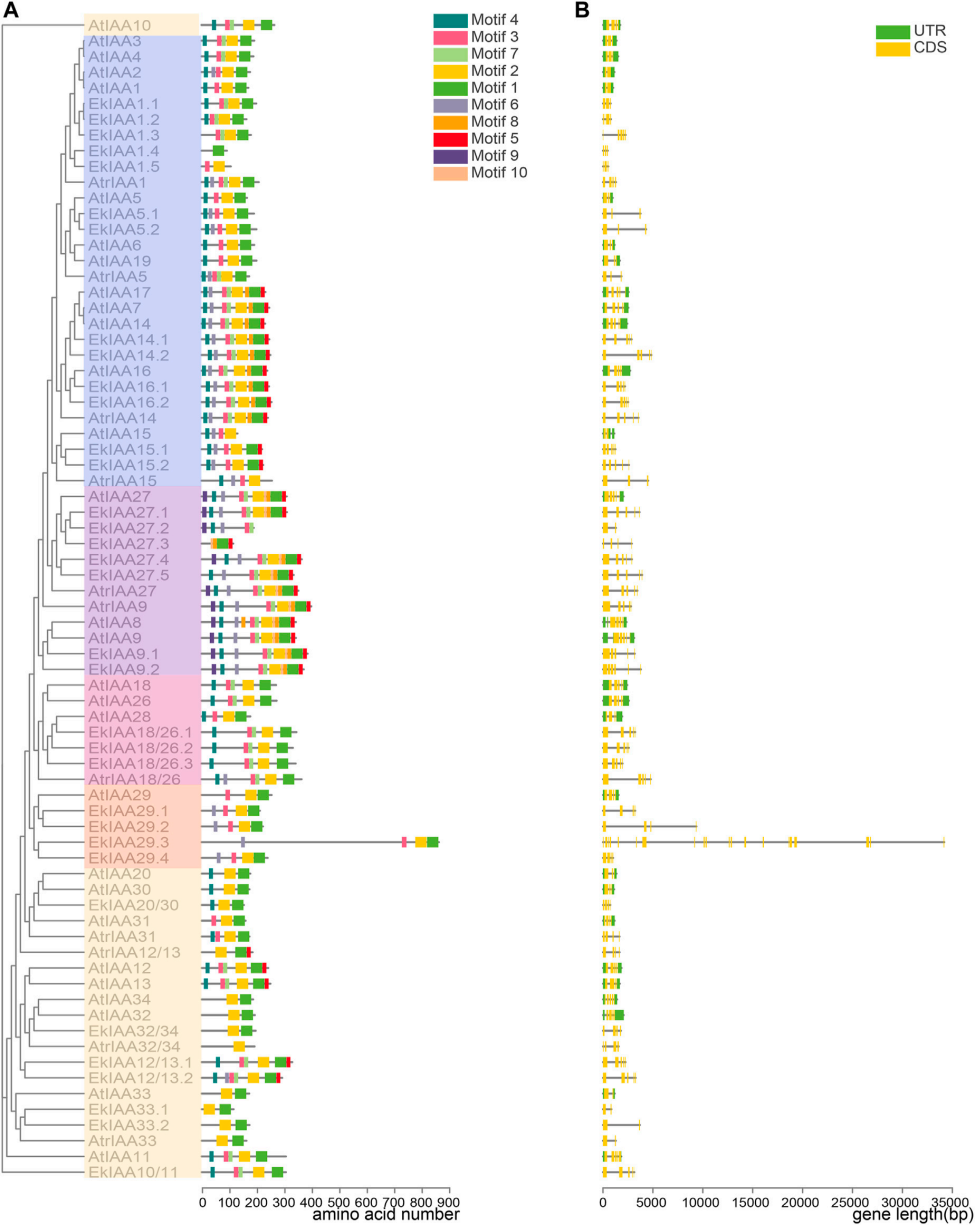
Gene structures of *EkIAA*s and conserved motifs in EkIAAs. **(A)** EkIAA phylogenetic tree and corresponding *EkIAA* gene structures. **(B)** Conserved protein motifs within EkIAAs. The phylogenetic tree was generated using the IQ-tree web server. Gene structures of *EkIAA*s were predicted with TBtools. The conserved motifs were analyzed using the MEME web server.

Protein motifs are critical for protein function and structure maintenance (Smith-Gill, 1991). Accordingly, we looked for conserved functional motifs in the predicted EkIAA and EkARF proteins with the MEME web server tool. We identified four domains conserved in EkIAA proteins (motifs 1–4), corresponding to IAA domains Ⅳ, Ⅲ, Ⅱ, and Ⅰ, respectively (Figure 4). Of the 34 EkIAA proteins, 23 (61.8%) contained all four conserved domains (domains I–IV) (Figure 4). Some EkIAA proteins lost one or more domains: For example, EkIAA1.3, EkIAA29.1, EkIAA29.2, EkIAA29.3, and EkIAA29.4 lack domain I; EkIAA20/30, EkIAA32/34, EkIAA33.1, and EkIAA33.2 lack domain II; EkIAA1.5 lacks domains I and IV; EkIAA27.2 lacks domains III and IV; and EkIAA1.4 and EkIAA27.3 have only domain I (Figure 4).

To understand the evolution of *EkIAA* genes, we examined their exon-intron structures (Figure 4A). Most *EkIAA* genes consisted of five exons and four introns, as in Arabidopsis, although introns in *EkIAA* genes were larger than those in their Arabidopsis counterparts (Figure 4). Three *EkIAA* genes (*EkIAA27.2*, *EkIAA33.1*, and *EkIAA33.2*) comprised only two exons and one intron, with another six EkIAA genes (*EkIAA1.1*, *EkIAA1.2*, *EkIAA1.4*, *EkIAA1.5*, *EkIAA5.1*, and *EkIAA5.2*) having three exons and two introns. Six *EkIAA* genes (*EkIAA20/30*, *EkIAA27.3*, *EkIAA29.1*, *EkIAA29.2*, *EkIAA29.4*, and *EkIAA32/34*) had four exons and three introns. *EkIAA9.2* had six exons and five introns, while *EkIAA29.3* had by far the most exons (20) and 19 introns (Figure 4). As the presence of conserved domains in the EkIAA proteins and the *EkIAA* gene structure are similar to those in their Arabidopsis orthologs (Remington et al., 2004), these results suggested that the *EkIAA* family in *E. konishii* is conserved.

We analyzed the conserved motifs and gene structure of EkARF proteins and *EkARF* genes, respectively (Supplementary Figure S1). EkARF proteins belonging to the same clade in the phylogenetic tree had the same functional motifs (Supplementary Figure S1). The DNA binding domain was represented by motifs 1, 9, and 10, while motifs 3, 6, and 8 matched the variable middle transcriptional regulatory region (MR). Motifs 7 and 5 formed part of the C-terminal dimerization domain (CTD) (Supplementary Figure S1). Of the 29 EkARFs, 21 (72.4%) contained all three functional domains, with only eight EkARFs (EkARF3, EkARF10*.*1, EkARF10.2, EkARF16.1, EkARF16.2, EkARF16.3, EkARF17.1, and EkARF17.2) lacking the CTD (Supplementary Figure S1). This variation in functional protein motifs may reflect mutations or deletions in the gene structure. Most *EkARF* genes contained 14 exons and 13 introns (Supplementary Figure S1). However, *EkARF17.1* consisted of only two exons and one intron, three *EkARF* genes (*EkARF16.1*, *EkARF16.2*, and *EkARF16.3*) were composed of three exons and two introns, *EkARF17.2* had five exons and four introns, and *EkARF3* contained 11 exons and 10 introns, which is consistent with the observed variation in protein domains (Supplementary Figure S1). Overall, *EkIAA* and *EkARF* genes appeared to be relatively conserved during evolution, but those derived from segmental duplication have experienced some structural divergence.

### *Cis*-element Analysis of *EkIAA* and *EkARF* Promoters

**FIGURE 5:**
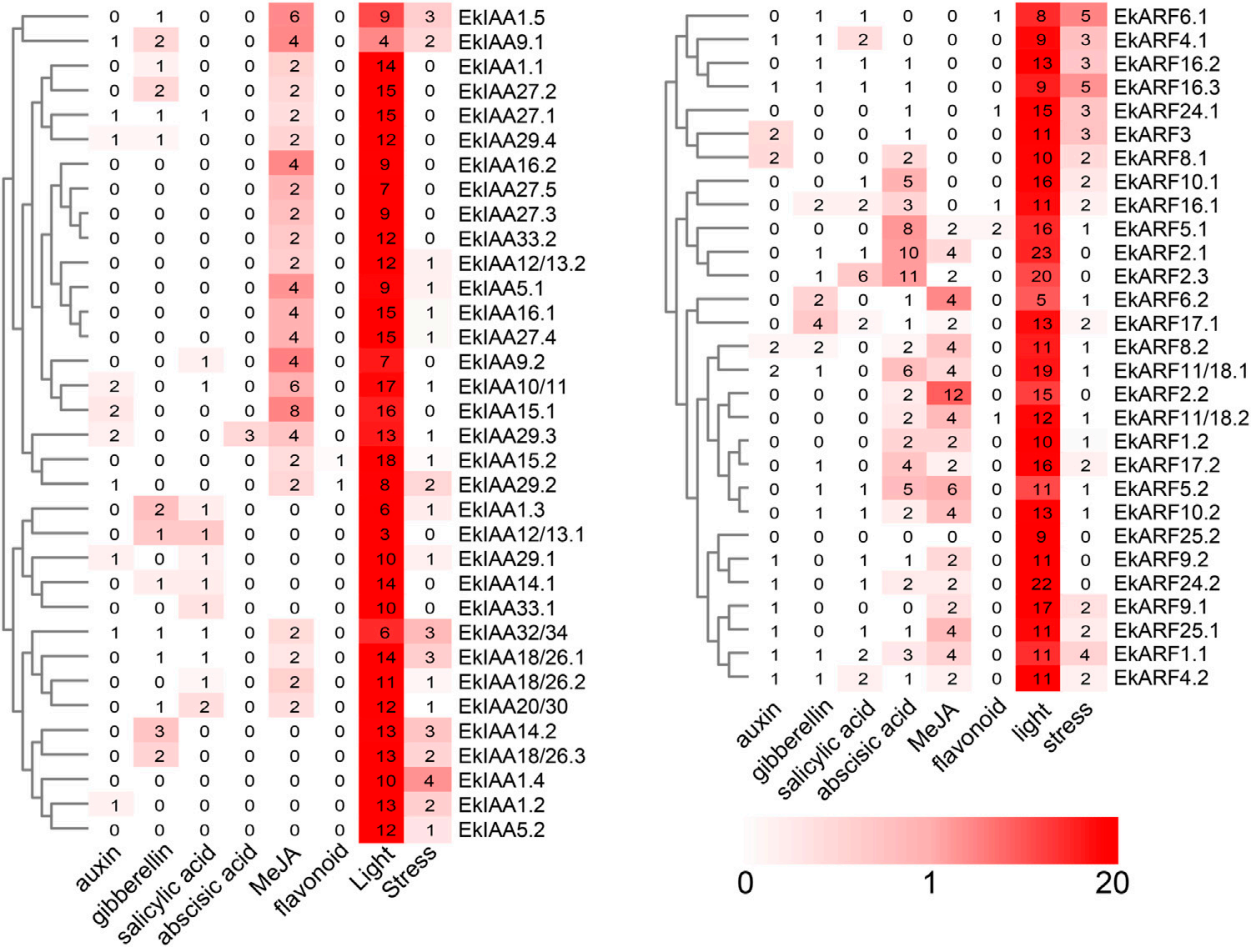
Predicted *cis*-elements in the *EkIAA* and *EkARF* promoters. The promoter sequences (−2,000 bp) of 34 *EkIAA* and 29 *EkARF* genes were analyzed by PlantCARE. The color bar indicates the number of *cis*-elements.

To explore the transcriptional regulation of *EkARF* and *EkIAA* genes and predict their functions, we analyzed the *cis*-regulatory elements in their promoters. We extracted 2,000 bp of upstream sequence, which we submitted to the PlantCARE online tool (Lescot et al., 2002). We then counted the number of phytohormone-, environment-, and flavonoid-responsive elements and noted their locations (Figure 5). *cis*-elements in *EkIAA* and *EkARF* promoters exhibited a similar pattern (Figure 5). Indeed, phytohormone-responsive and environmental stress–related *cis*-elements were present in all promoters of the *EkIAA* and *EkARF* gene family in *E. konishii*.

### Expression Patterns of *EkIAA* and *EkARF* Genes in *E. konishii*

**FIGURE 6:**
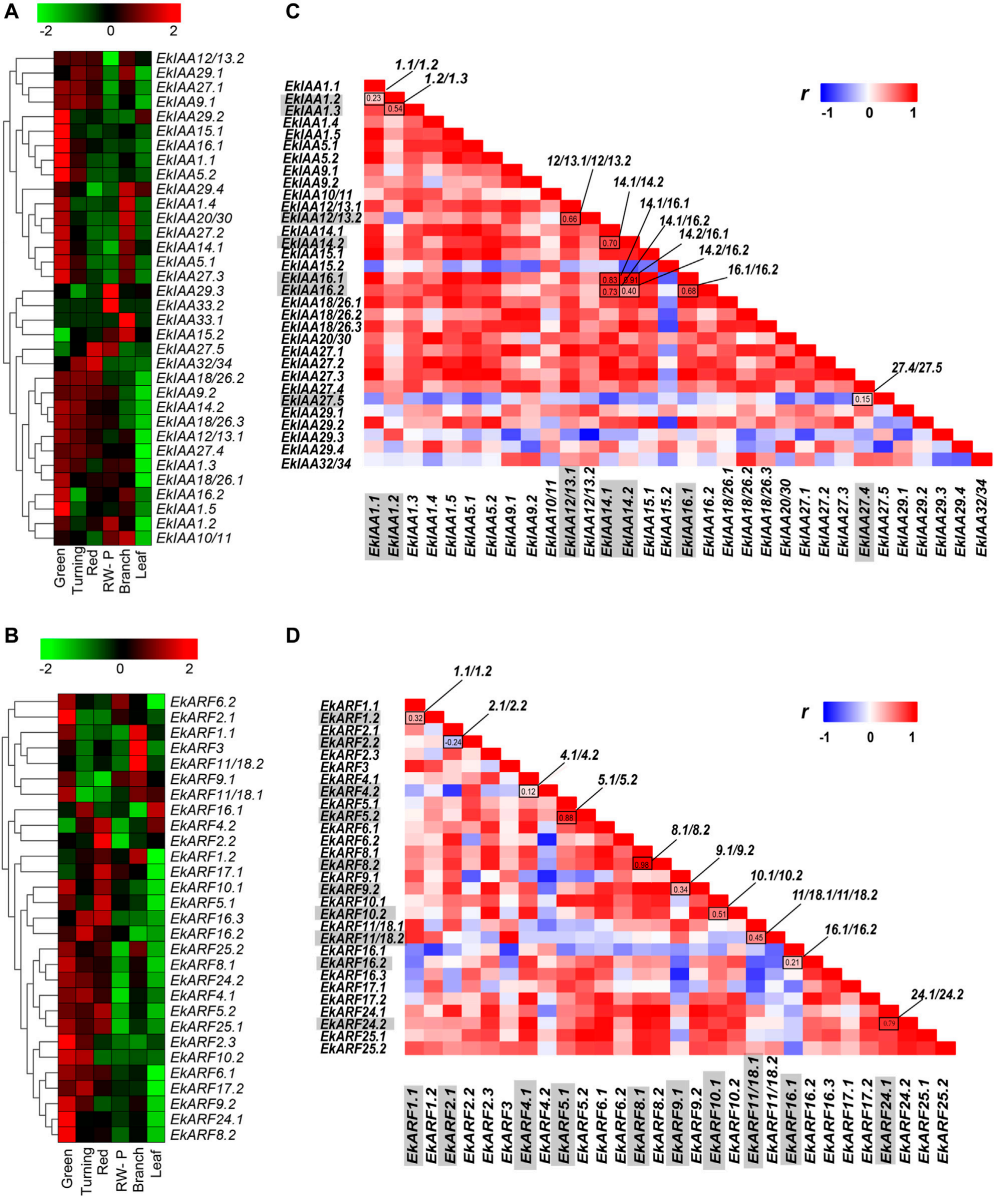
Heatmap representation of the expression of *EkIAA* **(A)** and *EkARF* **(B)** genes during fruit developmental stages (green, turning, red fruit, and red-winged pericarp) in branches and leaves. The color bar indicates Log_2_-normalized transcripts per million reads (TPM). RW-P, red-winged pericarp.

We then used publicly available RNA-seq datasets to analyze the expression patterns of *EkIAA* and *EkARF* genes at four fruit developmental stages, including green, turning red fruit, and red-winged pericarp stages, as well as in branches and leaves (Yang et al., 2020); the results are summarized as heatmaps in Figure 6. *EkIAA* gene family members showed varying expression patterns. Most *EkIAA* genes were highly expressed in green fruits, with the exception of *EkIAA15.2*, *EkIAA33.1*, *EkIAA33.2*, and *EkIAA29.3*, of which the first two were expressed specifically in branches, whereas the latter two genes were specifically expressed in red-winged pericarp (Figure 6A). *EkIAA27.5* and *EkIAA32/34* were highly expressed during the red fruit stage. *EkARF* genes were highly expressed during the fruit maturation stage (Figure 6B), of which *EkARF1.2*, *EkARF2.2*, *EkARF4.2*, *EkARF5.1*, *EkARF5.2*, *EkARF16.3*, and *EkARF17.1* showed high expression levels in the red fruit stage. Most *EkIAA* and *EkARF* genes were highly expressed in fruits, hinting at their potential involvement in fruit maturation, including the accumulation of associated secondary metabolites.

To assess the extent of functional diversification within these two families, we focused on duplicated gene pairs (10 *EkIAA* pairs and 10 *EkARF* pairs) and calculated the Pearson’s correlation coefficients of their expression profiles. Several duplicated gene pairs did in fact exhibit differential expression across the samples tested. Six *EkIAA* gene pairs (*EkIAA12/13.1/-12/13.2*, *EkIAA14.1/-14.2*, *EkIAA14.1/-16.1*, *EkIAA14.1/-16.2*, *EkIAA14.2/-16.1*, and *EkIAA16.1/-16.2*) and three *EkARF* gene pairs (*EkARF5.1/-5.2*, *EkARF8.1/-8.2*, and *EkARF24.1/-24.2*) showed similar expression patterns within the pairs, as evidenced by their high correlation coefficients (Figure 6B). We also identified one *EkIAA* pair and one *EkARF* pair with distinct expression levels between duplicated copies: *EkIAA27.5* (expressed at high levels in all samples) and the duplicated copy *EkIAA27.4* (expressed at relatively low levels), with a correlation coefficient of 0.15 (Figure 6C); and *EkARF11/18.1* (expressed at low levels in all tissues) and *EkARF18.2* (highly expressed in branches), with a correlation coefficient of 0.45. (Figure 6D). These results suggested that the functions of duplicated genes may have diverged following the initial duplication event.

### EkARFs May Regulate the Biosynthesis of Terpenoids and Anthocyanins

**FIGURE 7:**
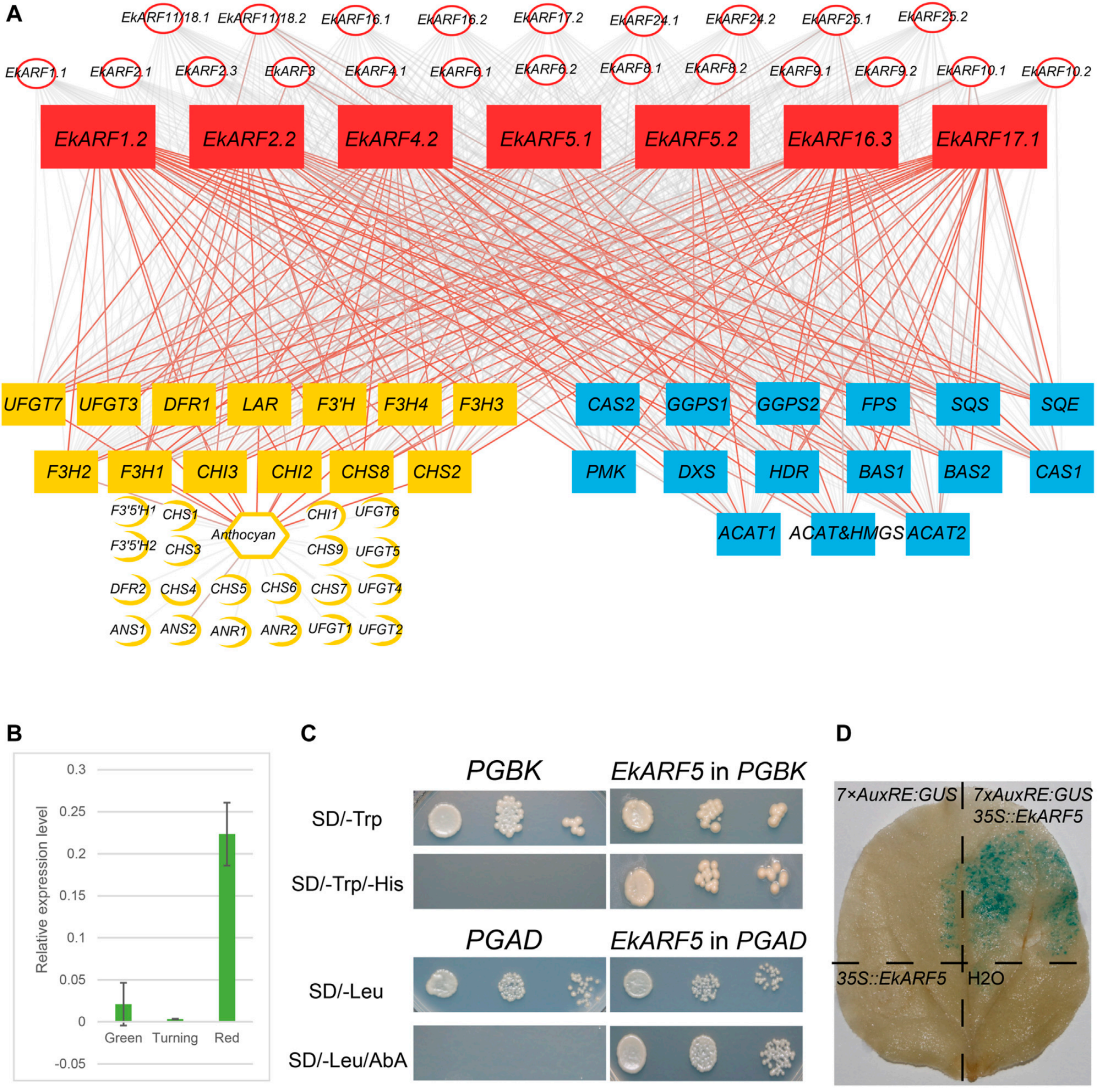
Correlation analysis between anthocyanin contents and *EkARF* expression, and triterpenoid contents and *EkARF* expression. **(A)** Red, *EkARFs*; yellow, anthocyanins; blue, triterpenoids. The gray lines indicate weak correlation, and red lines indicate strong correlation. **(B)** RT-qPCR results. **(C)** Yeast one-hybrid analysis results. **(D)** Results from GUS staining.

Medicinal compounds such as triterpenes, phenolic acids, and flavonoids have been isolated from *Euscaphis* fruits, leaves, and roots (Liang et al., 2018). The accumulation of anthocyanin and terpenoid secondary metabolites coincides with *E. konishii* fruit maturation (Yuan et al., 2018b; Liang et al., 2019). Generally, genes with similar expression patterns may have related roles as they belong to the same regulatory pathway or are regulated by the same upstream factors. Thus, co-expression detected from our correlation analyses may provide cues as to gene regulation or function. Because anthocyanin contents, and the key anthocyanin biosynthetic genes, were previously well characterized during *Euscaphis* fruit development (Yuan et al., 2018b), we performed a correlation analysis between anthocyanin levels and the expression estimates for anthocyanin biosynthesis genes, *EkIAA*, and *EkARF* genes. We determined that anthocyanin contents were positively and strongly correlated with the expression of five genes encoding key enzymes (*CHALCONE SYNTHASE8* [*CHS8*], *CHALCONE ISOMERASE2* [*CHI2*], *FLAVANONE 3-HYDROXYLASE1* [*F3H1*], *F3H2*, and *F3H3*) and very strongly correlated with that of another eight genes (*CHS2*, *CHI3*, *F3H4*, *FLAVONOID 3′-HYDROXYLASE* [*F3′H*], *DIHYDROFLAVONOL 4-REDUCTASE1* [*DFR1*], *LEUCOANTHOCYANIDIN REDUCTASE* [*LAR*], *FLAVONOL 3-O-GLUCOSYLTRANSFERASE3* [*UFGT3*], and *UFGT7*) (Figure 7A). Because ARF proteins regulate the expression of their target genes by binding to their cognate *cis*-elements in promoters, we looked for *EkARF* genes co-expressed with the anthocyanin biosynthetic genes listed above (Figure 7A), leading to the identification of seven such genes (*EkARF1.2*, *EkARF2.2*, *EkARF4.2*, *EkARF5.1*, *EkARF5.2*, *EkARF16.3*, and *EkARF17.1*).

Triterpenoids accumulate to high levels in *Euscaphis* fruits and are important raw materials for natural products, food additives, and chemical products. Genes encoding the enzymes involved in the biosynthesis of triterpenoids have been described in the *Euscaphis* genome (Huang et al., 2019; Liang et al., 2019), prompting us to test for correlations between their expression patterns and those of *ARFs* genes (Figure 7A). This analysis highlighted seven *EkARF* genes (*EkARF1.2*, *EkARF2.2*, *EkARF4.2*, *EkARF5.1*, *EkARF5.2*, *EkARF16.3*, and *EkARF17.1*) whose expression was positively and strongly correlated with genes involved in triterpenoid accumulation (Figure 7). These results suggested that *ARF* genes contribute to secondary metabolite biosynthesis in *Euscaphis*.

ARF proteins are transcription factors that can bind to AuxREs (TGTCTC) to regulate the expression of their target genes. We noticed at least one AuxRE either upstream, downstream, or within intronic regions of anthocyanin and triterpenoid biosynthetic genes (Supplementary Table S3). To assess the role of ARFs in anthocyanin and triterpenoid biosynthesis, we selected *EkARF5.1* for further characterization. RT-qPCR showed that *EkARF5.1* is highly expressed during the red fruit stage, which is consistent with the RNA-seq data (Figure 7B). We fused EkARF5.1 to the GAL4 activation domain (AD) (AD-EkARF5.1) and introduced the resulting construct into yeast strain Y1HGold carrying a reporter consisting of seven copies of the AuxRE sequence driving the expression of *Aureobasidin Resistance 1*, conferring resistance to the antibiotic aureobasidin A (AbA). Whereas yeast colonies carrying AD or AD-EkARF5.1 grew on synthetic defined medium (Figure 7C), only yeast cells harboring the *AD-EkARF5.1* construct survived growth on AbA-containing medium (Figure 7C), supporting the notion that EkARF5.1 binds to the AuxRE. We then tested the transactivation activity of EkARF5.1 by fusing full-length EkARF5.1 to the GAL4 DNA binding domain (BD) to generate the BD-EkARF5.1 fusion protein; the resulting encoding construct was introduced into yeast strain Y2HGold, which harbors the *His3* gene driven by a GAL4-responsive promoter (Figure 7C). Only yeast cells carrying the *BD-EkARF5.1* construct survived on synthetic medium lacking histidine, unlike yeast cells carrying the empty GAL4 DB vector (Figure 7C). These results indicated that EkARF5.1 has transactivation activity in yeast cells. Finally, we tested EkARF5.1 in *N. benthamiana* leaf epidermal cells by co-infiltrating a construct overexpressing *EkARF5.1* and a *β-GLUCURONIDASE* (*GUS*) reporter construct whose expression is driven by seven copies of the AuxRE. We detected GUS activity in plant cells only when *EkARF5* was co-expressed (Figure 7D). These results demonstrate that EkARF5.1 may play a role during anthocyanin and triterpenoid biosynthesis.

## Discussion

Plant secondary metabolites consist of various bioactive compounds with applications in medicine and industry. The biosynthesis of plant secondary metabolites is regulated not only by plant growth and development signaling, but also by environmental stress cues. In China, *E. konishii* is widely planted as a medicinal and ornamental plant, but the regulatory mechanism of secondary metabolite biosynthesis is poorly understood, which limits genetic improvement and development of agronomic management techniques. In this study, we identified 34 *Aux/IAA* genes and 29 *ARF* coding sequences that map to some, but not all, linkage groups that constitute the *E. konishii* genome. Co-expression analysis suggested that seven *EkARF* genes may regulate anthocyanin and triterpenoid biosynthesis in *E. konishii*. Our data improve the understanding of the *EkIAA* and *EkARF* gene families and may provide valuable information on their biological functions in the context of secondary metabolite biosynthesis.

Auxin is an essential plant hormone, and Aux/IAAs and ARFs are key components of the signaling transduction process. Most of the current knowledge on Aux/IAA and ARF function, gene expression, and regulation has been obtained from studies in annual herbaceous plants such as Arabidopsis, rice, and tomato (Luo et al., 2018), while much less is known about IAAs and ARFs in longer-lived species, such as the evergreen shrub *E. konishii*. In this study, we identified 34 *Aux/IAA* and 29 *ARF* family members in the *E. konishii* genome (Figure 1; Supplementary Table S1), which was comparable to the numbers in other species, such as Arabidopsis (29 *Aux/IAAs* and 23 *ARFs*) (Remington et al., 2004), poplar (35 *Aux/IAAs* and 39 *ARFs*) (Kalluri et al., 2007), maize (31 *Aux/IAAs* and 31 *ARFs*) (Wang et al., 2010), and rice (31 *Aux/IAAs* and 25 *ARFs*) (Jain et al., 2006). *EkIAA* and *EkARF* genes clustered into five groups, as previously reported in Arabidopsis (Remington et al., 2004). Most *EkIAA* and *EkARF* genes within the same phylogenetic group shared similar exon-intron structures and the same arrangement of functional motifs in their encoded proteins (Figure 4), likely reflecting the gene duplication events that have shaped the expansion of the *EkIAA* and *EkARF* gene families in the *E. konishii* genome. *K*_a_ */K*_s_ values of homologous genes further showed that duplicated genes underwent purifying selection (Supplementary Table S1). These results indicated that the two gene families are evolutionarily conserved with those from other plant species and may thus exhibit the same function and biochemical characteristics in *E. konishii.* However, we also identified six EkIAAs (EkIAA1.3, EkIAA29.1, EkIAA29.2, EkIAA29.3, and EkIAA29.4) that lack domain I (Figure 4), which are not expected to repress their downstream targets because domain I can repress the expression of target genes when in close proximity to the promoter (Hagen and Guilfoyle, 2002). Therefore, the *E. konishii* genome encodes conserved *EkIAA* and *EkARF* gene family members, although some members exhibit domain loss, possibly having arisen from unknown segmental duplication events, which will increase the complexity of auxin regulation.

The phytohormone auxin plays critical roles during plant growth. During fruit development, auxin also induces fruit set and growth, whereas it represses fruit maturation and ripening (Pattison et al., 2014). During tomato fruit maturation, the *SIARF2* expression level increases in response to stimulation by ethylene, suggesting that auxin may repress fruit ripening (Pattison et al., 2014). Although gene structure and their encoded protein motifs were conserved in *EkIAA* and *EkARF* gene families, a subset of *EkIAA* and *EkARF* genes showed high expression during fruit maturation and ripening (Figure 6). Seven *EkARF*s were highly expressed during fruit maturation, of which *EkARF1.2*, *EkARF2.2*, *EkARF4.2*, *EkARF16.3*, and *EkARF17.1* encode proteins containing a proline/serine/threonine-rich domain that acts as a transcriptional repressor (Supplementary Figure S1) (Tiwari et al., 2003). These results suggested that *E. konishii* fruit maturation and ripening may be similar to tomato. Interestingly, the two ARF5 homologs EkARF5.1 and EkARF5.2 contained a glutamate-rich domain that functions as a transcriptional activator domain; their encoding genes were highly expressed during *E. konishii* fruit maturation and ripening (Supplementary Figure S1) (Tiwari et al., 2003; Guilfoyle and Hagen, 2007), indicating that auxin signaling may play distinct roles during *E. konishii* fruit maturation and ripening processes. In Arabidopsis, ARF5 affects meristem development (Dastidar et al., 2019), while we showed here that *EkARF5* is highly expressed during fruit maturation and ripening, likely reflecting changes in the promoter region associated with *E. konishii* and suggesting that the EkARF family underwent subfunctionalization during its evolutionary history. The high accumulation of secondary metabolites is a main feature of *E. konishii* fruit maturation and ripening (Yuan et al., 2018b). Co-expression analysis further revealed that *EkARF5.1* and *EkARF5.2* expression is positively and strongly correlated with that of anthocyanin and triterpenoid biosynthetic genes (Figure 7A). We confirmed that *EkARF5.1* is highly expressed during fruit maturation and ripening stages and that EkARF5.1 can directly bind to AuxREs located within the promoter regions of anthocyanin and triterpenoid biosynthetic genes to activate their transcription in yeast and plant cells (Figures 7C,D). These results strongly suggest that EkARF5.1 and EkARF5.2 may be positive regulators of secondary metabolite biosynthesis, although the exact mechanisms by which they regulate anthocyanin biosynthesis require further study.

*E. konishii* fruit maturation and ripening involve pericarp splitting, pericarp overturn, and the accumulation of secondary metabolites (Yuan et al., 2018b; Liang et al., 2019; Huang et al., 2019; Sun et al., 2021). Pericarp overturn, the process in which the pericarp morphological changes after pericarp splitting (Supplementary Figure S2), is the main difference between *E. konishii* fruits and those of grape (*Vitis vinifera*) and tomato (Yuan et al., 2018b; Liang et al., 2019; Sun et al., 2021), which and may contribute to its survival and enable expansion to new environments (Sun et al., 2021). Pericarp overturn may be the result of uneven cell growth between epicarp and endocarp (Sun et al., 2021), which is associated with cell growth or differentiation (Ding et al., 2011). Auxin asymmetric distribution mediated by development and environmental cues results in uneven cell growth, thus regulating plant growth and response to environmental changes (Ding et al., 2011). Therefore, the highly expressed EkARF5s are probably involved in pericarp overturn in the last stage of *E. konishii* fruit maturation (Figure 6B; Figure 7B). It has been reported that auxin or its signaling is involved in biosynthesis of secondary metabolites such as anthocyanin, flavonols, and glucosinolates (Lewis et al., 2011; Wang et al., 2018; Wang et al., 2020). Given that pericarp split, pericarp overturn, and secondary metabolites are coupled (Yuan et al., 2018b), they may be regulated by similar or identical molecular mechanisms. In our study, we identified AuxREs in the promoters of anthocyanin and triterpenoid biosynthetic genes (Supplementary Table S3), to which EkARF5 can bind in yeast and plant cells, suggesting that EkARF5-mediated auxin signaling may regulate multiple signaling pathways in *E. konishii* fruit maturation.

## Conclusion

We comprehensively analyzed the *Aux/IAA* and *ARF* gene families in *E. konishii*, which are evolutionarily well conserved. Expression and co-expression analyses showed that EkARF5 may play critical roles during the regulation of secondary metabolite biosynthesis. This study provides the basis for uncovering the regulatory mechanisms necessary to boost the production of industrial products and breed new *E. konishii* varieties with high economic output.

## Data Availability

Publicly available datasets were analyzed in this study. This data can be found here: The E. konishii chromosome-level genome assembly and annotation data (Accession No. GWHBCHS00000000) were available from National Genomics Data Center (https://ngdc.cncb.ac.cn/gwh/Assembly/reviewer/DsVzSvoOmVHEsqvwRYFCzmaHLYVsIsbpuBGJNDyDMGIWICZvIzpChREHnNqERbWc).

## Abbreviations

AbA, antibiotic aureobasidin A; AD, activation domain; ARF, auxin response factor; Aux/IAA, auxin/indole-3-acetic acid; AuxRE, auxin response element; BD, binding domain; CTD, C-terminal dimerization domain; DBD, DNA-binding domain; GA, gibberellic acid; HMM, Hidden Markov model; LG, linkage group; Ka, number of nonsynonymous substitutions per nonsynonymous site; Ks, number of synonymous substitutions per synonymous site; MR, middle region; pI, isoelectric point; SD, synthetic defined.
