# Supplementary figures and images for: Genome-Wide Identification and Co-Expression Analysis of ARF and IAA Family Genes in Euscaphis konishii: Potential Regulators of Triterpenoids and Anthocyanin Biosynthesis
Source: Front Genet. 2022 Jan 5;12:737293. doi: 10.3389/fgene.2021.737293 (PMC8766721; doi:10.3389/fgene.2021.737293)

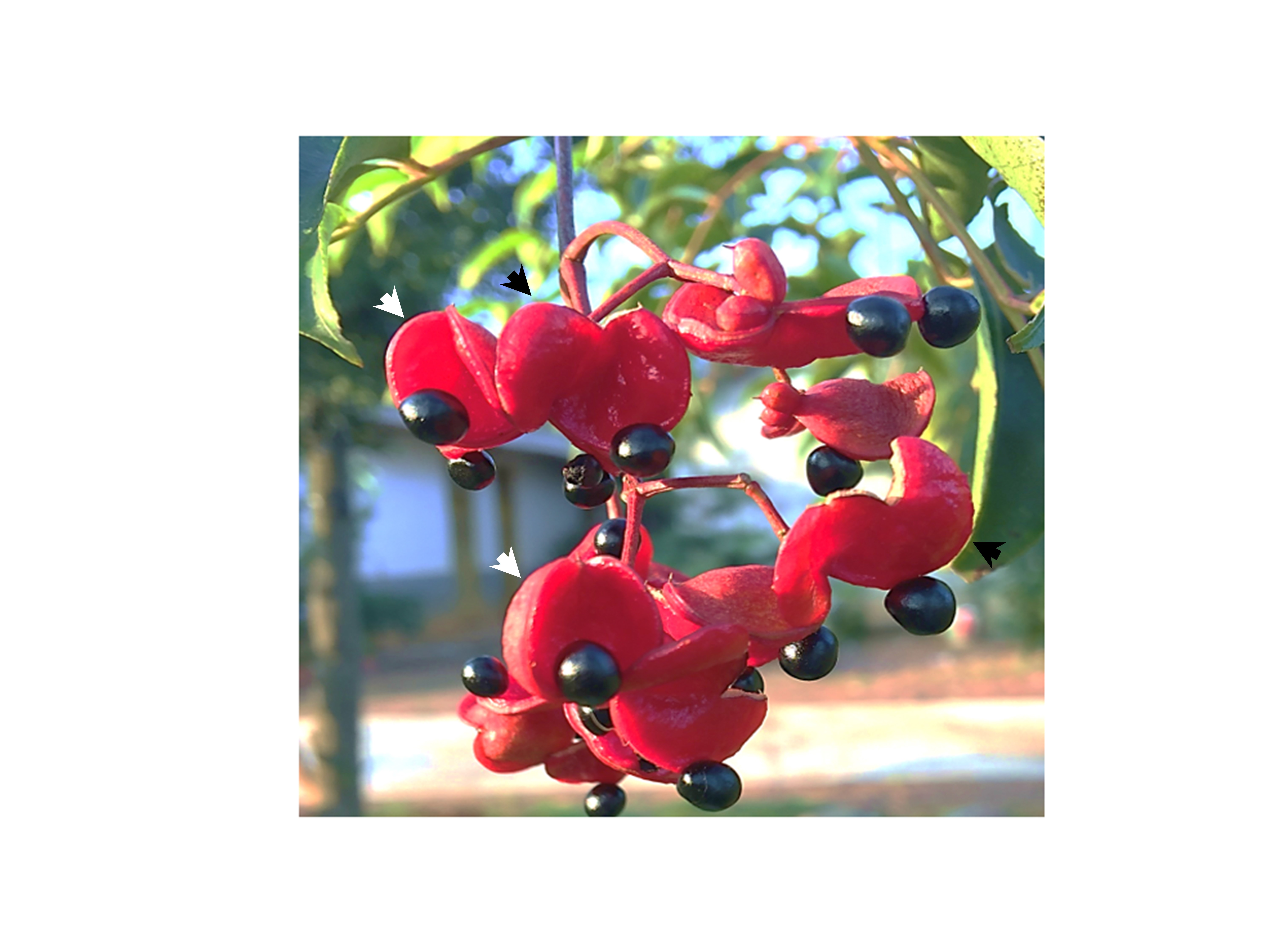

Supplement: Supplementary file 1 [file Image1.JPEG]

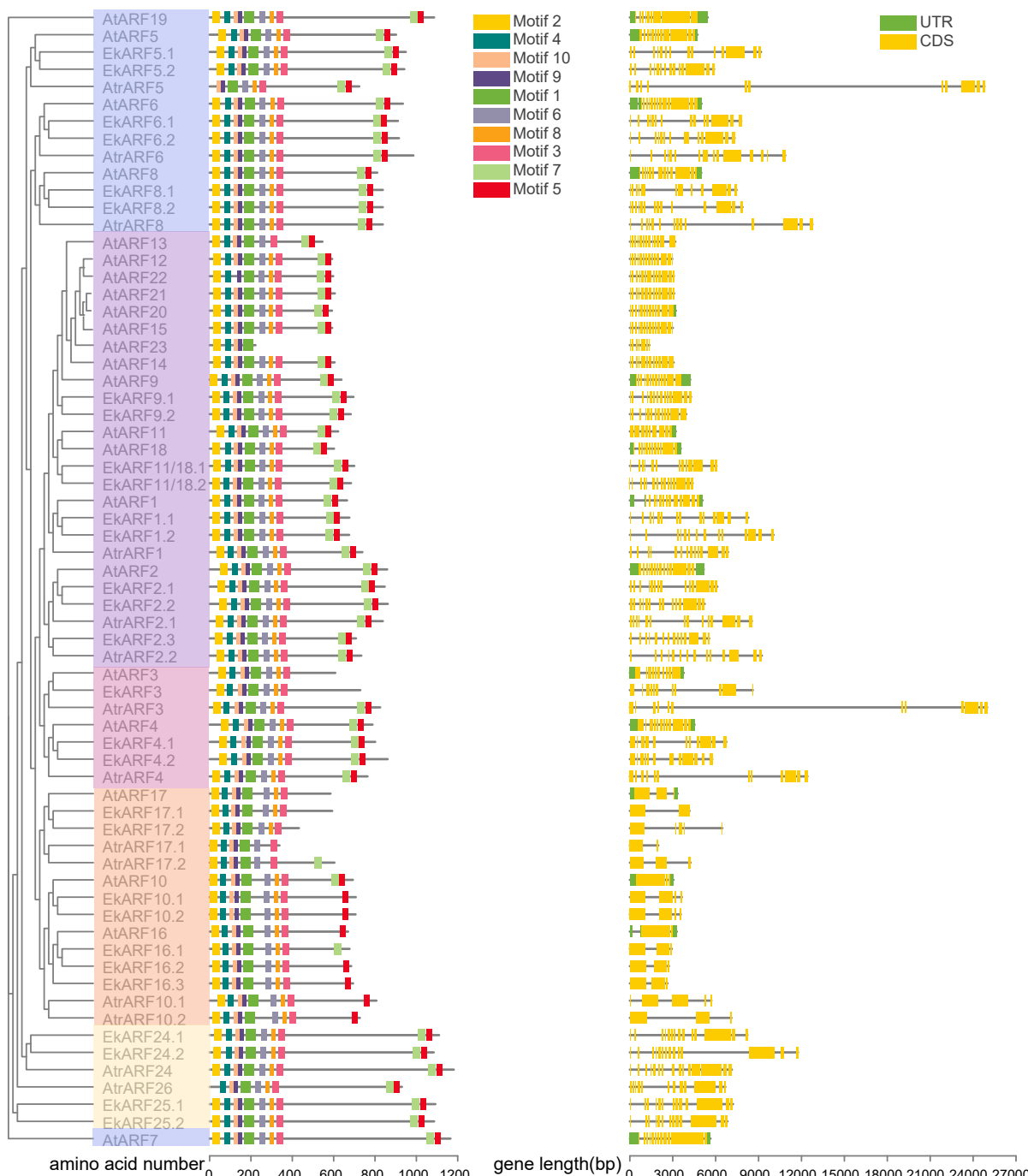

Supplement: Supplementary file 2 [file DataSheet1.PDF]
